# The Scientific Spirit and the Ethics of Dental Practice

**Published:** 1895-06

**Authors:** C. J. Essig

**Affiliations:** Philadelphia


### THE SCIENTIFIC SPIRIT AND THE ETHICS OF DENTAL PRACTICE.[1]

BY DR. C. J. ESSIG, PHILADELPHIA.

"The individual," some one has said, "is a puzzle; in the aggregate he becomes a mathematical certainty. You cannot foretell what one man will do, but you can say with precision what an average number will be up to." In no calling is this more conspicuously true than in the dental profession; its rapid advancement, its triumphs and scientific spirit, very justly entitle it to the admiration and pride of its members. Yet among dentists it is often asserted that there is no *esprit de corps*, and the frequent instances of disregard of the code of ethics on the part of individuals, and the unscientific spirit in which many of them view the lesions of the organs which it is their specialty to treat, make a bold contrast between the high aims of the profession as a whole and the practice of a large number of its members.

In our societies, current literature, and colleges, in the free and liberal professional intercourse which has been steadily growing for more than a generation, may be found the most gratifying improvement over the time when the tendency was to conceal every invention, device, or detail of practice, as the miser does his gold.

It cannot be denied, however, that *esprit de corps*, which is the "chivalry" of professional life, is often forgotten, and the code of ethics which should be our guidance in all intercourse, both with patient and professional brother, is as often ignored as observed. Yet we should be far from attributing every violation of the strict interpretation of the code of ethics to a want of honor or to greed; much of it is no doubt due to absence of the scientific spirit, which should guide the practitioner in the treatment of the dental organs.

The word "scientific" is often used by dentists in their writings and discussions, and they not infrequently misapply the term. Dentistry as practised is an art, even when the practitioner considers his filling-materials as therapeutic agents, therapeutics being itself essentially the art of medicine. A science teaches us to know, an art to do; with the light, then, which a knowledge of the so-called medical sciences sheds upon what we do, our work should be better done, and with greater uniformity in methods and results,

---

[1] Read before the Academy of Stomatology, Philadelphia, April 23, 1895.

than has yet been attained in the prevention or treatment of dental caries.

The remarkable want of concordance in diagnosis and in treatment in dental practice; the absence of *esprit de corps* and indifference to the ethics of questions of judgment between dentists, especially when, as is too often the case, the patient is made a party to controversies growing out of difference in treatment, has undoubtedly been a serious injury to our profession in the estimation of the public. The dental profession cannot hope to gain or to hold the highest respect of the people while its individuals exhibit the ethics of the artisan.

This fault comes to us from a variety of causes; primarily we get it as an inheritance from predecessors who flourished previous to the advent of dental colleges; they were often mere mechanics, without knowledge of the medical sciences, and their unwritten ethics were those of the artisan. Another cause may be found in the fact that filling teeth with gold is artistic in character, requiring the artistic instinct in its performance. The practitioner is thus lured from the scientific aspect of his specialty, which otherwise might stimulate him to find, in the realms of preventive medicine, lines of treatment of a higher order than the merely mechanical and often unsatisfactory one of filling carious cavities with gold, amalgam, or the "plastics." Another cause, which is now happily growing less every year, may be found in the fact that many recruits enter the ranks of the dental profession after having passed a portion of their earlier lives in commercial pursuits, and they bring with them the ethics and ideas of the shop, where the ability to sell often consists in the power to underestimate the wares of a rival. Such men are not likely to get beyond the idea of mechanical achievement in the treatment of dental organs.

Every dentist owes something to his profession, which freely lays its treasures at his feet, and if he is not endowed with sufficient self-respect to avoid practices which will dishonor him, he should be compelled to respect that profession whose sheltering wings include him in their fold, and whose fair fame may be smirched by every mean act of his professional life.

I was asked not long since, by a legal gentleman, the question, "Is there no *esprit de corps* in your profession?" Said he, "A relative of mine whose teeth had just been examined by a dentist of high standing, and pronounced in good order, called upon another member of your profession in a neighboring town, who claims to have found a large amount of work to be done, and has so shaken the confidence of my relative that he and some of his family have left their family dentist." Now, said he, "If this incident merely indicates an honest difference of judgment, then there must be some serious defect in your system of professional education; but if this difference of opinion is, as I suspect, actuated by a commercial spirit, there should be a penalty made to follow such unprofessional conduct severe enough to make it unprofitable for him to repeat it."

I have heard incidentally of a case which occurred recently, of a dentist who made an examination of fillings done by a previous dentist in the presence of two of the patient's relatives, pointing out alleged defects, and using other arguments to destroy the confidence which they felt for many years in their family dentist. I was asked by the lady who related this circumstance to me, "Does your profession allow such conduct?" I replied that "our profession prescribed for its members a code of ethics for their guidance, but that no written 'code' could make a gentleman of an individual who was not so naturally or by training."

A gentleman asked me recently for an opinion about the case of an acquaintance who had his teeth examined by a dentist who told him that it would take six weeks to put them in order; he kept appointments every day for three weeks, when, getting "a little tired," he asked the dentist how much more remained to be done, and was told, "I am about half through." "Well," said the patient, "I think I will stop for a while." But soon after that he had occasion to consult another practitioner, who, after an examination, told him, that with the exception of trifling repairs needed in one tooth, his teeth were in perfect order. I prefer not to characterize this remarkable difference of opinion, but presuming that the incident was one of an honest difference of judgment between two professional men of equal intelligence, skill, and training, how may we account for such diagnostic uncertainty. The public is, I believe, more and more coming to attribute such differences to the commercial spirit, and thus our profession is, to a certain extent, made to rest under a stigma.

Viewing dentistry as a branch of the healing art, examinations made to determine which teeth are carious and in need of treatment correspond to diagnosis in medicine, being, however, much simpler in character and easier to determine; yet medical diagnosis has become almost an exact science, while dental diagnosis, among a very large number of educated and prominent individuals often making pretentious claims to high professional attainment, remains so uncertain that it must inevitably bring distrust and ridicule upon our profession.

An example of individual disregard for professional ethics, and of the greed which often overrides every other consideration, was related to me by one of my patients, who stated that her sister's dentist had removed every filling in her mouth done by her two preceding dentists, and is now removing his own fillings.

The use of gutta-percha and other plastic filling-materials affords the unscrupulous individual his best opportunity for unprofessional criticism. A dentist who has been in continuous practice for more than half a century recently remarked to me, that "the most discouraging feature which he had met with in practice was to be found in those cases in which he had endeavored to save the soft and inferior teeth of early childhood by filling with gutta-percha and other plastic materials, and just when the age had been reached when sufficient improvement in the quality of such teeth seemed to warrant the use of gold with some degree of permanency, they would fall into the hands of some other dentist, presumably through loss of confidence in the family dentist; for some patients think that, unless gold is used from the first as a filling-material, we have not used our best efforts, and will seek the services of some one else, who, perhaps, will be only too ready to encourage the waning confidence in his professional brother. It is in just such cases that the individual finds his most prolific opportunity; he gets a patient of fifteen or sixteen years of age, in whose mouth, during early childhood, the teeth were of the frailest character, in some cases, perhaps, showing decalcification of the enamel on the labial surfaces of both upper and lower incisors. No class of cases cause the earnest practitioner more anxiety than do these; it would seem almost like malpractice to attempt to bridge over that precarious period of early dentition with any other of our too few and often unsatisfactory filling-materials than gutta-percha. But if the patient is unlucky enough to get into the hands of a man who habitually ignores the ethical side of cases, he frowns, looks unutterable things, condemns the earlier treatment, and fills with gold, the very thing the family dentist would have done at that time had the patient remained in his charge, for, by the fifteenth to the seventeenth year, the teeth often show great improvement, and sometimes the tendency to decay at that age will have entirely disappeared.

Ten years ago one of my young patients, who had teeth of the frailest character, so frail, indeed, that up to his fourteenth year I

believed that I could better arrest the progress of decay by the use of gutta-percha, got into the hands of a dentist at one of the fashionable watering-places, who, between himself and his assistant, after much severe and out-spoken criticism, replaced all the gutta-percha fillings with very inferior gold ones, yet that individual has, I believe, on more than one occasion publicly advocated the use of gutta-percha as a preliminary filling-material in "children's teeth of the frailest class."

Three or four years ago a striking instance of individual disregard for the code of ethics, which our profession prescribes for its members, came to my knowledge in the case of a young miss whose teeth were of very inferior quality, and for whom, up to her fifteenth year, I did not dare to use any other filling-material than gutta-percha. Having some discomfort with a very frail upper molar, she was taken in my absence, during the summer vacation, to another dentist, who extracted the first molar, and with many expressions of horror hastened to replace with gold what gutta-percha fillings he found in the front teeth. Now, it happened that in this case I had special reasons for wishing to preserve the first superior molar on account of a flat appearance of the face, which I feared would be greatly increased by the loss of that tooth, but when I again saw the patient in the autumn the mischief had been done; the scientific idea which should have governed in the treatment of the case had given place to the spirit of greed. The loss of the molar tooth, with the subsequent increased flattening of the face and the change in the centre line, was a matter of much regret to the child's parents, who were in no way responsible for the unwarrantable interference of the dentist, to whom the child was taken for some trifling repair to the tooth which was extracted.

Seventeen years ago I made an examination of the teeth of a lad of nineteen. I found a full denture of superior quality without caries, and so informed the boy's mother, at which she expressed surprise, and told me that within ten days her son's teeth had been examined by a dentist who recorded, as a result of that examination, "twenty small cavities." That patient is now thirty-six years of age. I have examined his teeth every six months since, and he has at present only two small gold fillings in his mouth. In this case I would not like to attribute the defect in the first diagnosis to other than one of judgment, but the patient becomes, necessarily, a party to the question, and whether the verdict of the public is that such remarkable differences are merely matters of opinion or exhibitions of the commercial spirit, the effect is equally discrediting to the dental profession as a whole.

Now, it may be asked, Have you not overestimated this matter? Have you not cited a few exceptional cases? And do not other professions do these things too? I believe that violations of the code of ethics are practised to a greater extent than I have herein pictured. I have not selected as examples of this disagreeable feature of our profession a few exceptional cases. I have presented what I believe to be a few typical cases of practices which we all know do exist.

It cannot be denied that other professions have their "black sheep," and that habitual violations of the "code" to a certain extent is practised in the medical profession, but it is not as outspoken or as flagrant, and is confined more to the lower stratum of its members than it is in the dental profession, and is more severely dealt with when detected.

I imagine the remedy for abuses which have doubtless greatly retarded free and full association among dentists, and to a certain extent lessened the confidence of the public in our profession, will be found not so much in the arraignment of individuals as in improvement of methods. We still estimate gold as the best material for filling teeth, yet it is a significant fact that the best examples of gold fillings are seen in the teeth of young adults or those who have passed the age when fillings are most imperatively needed, and that gold is often the most unfit material with which to arrest caries of the frailest teeth, particularly those of early childhood, at the very time when effective measures are most demanded.

Neither from the prophylactic nor therapeutic stand-points has dental caries been sufficiently studied as a disease. Dentists have been so engrossed in the artistic treatment of the teeth, and the development of mechanical appliances, that a rational systemic treatment has been almost entirely overlooked. Caries of the teeth may with as much propriety be considered a disease as is "Bright's" or any other lesion affecting the different organs of the body, yet any of these may be controlled or cured by medicinal or hygienic treatment.

A properly regulated diet, with systematic physical exercise for the purpose of promoting assimilation to the extent demanded by nature for a normal condition of all the organs of the body, would do much to control and even cure dental caries. Many of our young patients have really no physiological right to have teeth, so perverted are the nutritive functions of their bodies. Does it not, then, seem that a more rational treatment would consist in a hygienic system which would so build up the tooth-structure that it would be capable of retaining the mechanical stoppings over which we spend so much time, skill, and patience, and that would reduce the chances of a recurrence of caries to a minimum?

In the fact that the field-hands among the Southern negroes have exceptionally fine teeth, while the house-servants show rapid deterioration of the dental organs, and in the superiority of quality of the teeth of the laboring classes of Europe over those of the non-laboring residents of cities, may be found significant and useful hints in the treatment of these organs.

In addition to a well-thought-out systemic treatment, a filling-material is needed which shall require less time and skill in its application than does gold, and which, when applied, shall possess the qualities of impermeability, insolubility, and a close resemblance to the enamel and dentine of the natural tooth; be non-conductive of thermal changes, and, above all, one that can be inserted in a plastic state, with the ability to fully harden afterwards.

That both of these improvements in the treatment of diseases of the teeth will eventually come I do not for a moment doubt; and when it does, the scientific spirit will, it is to be hoped, take the place of the idea which leads so many dentists to believe that a well-finished gold filling is *per se* the highest aim of conservative dentistry.

